# “Thanks for Letting Us All Share Your Mammogram Experience Virtually”: Developing a Web-Based Hub for Breast Cancer Screening

**DOI:** 10.2196/cancer.8150

**Published:** 2017-10-27

**Authors:** Adam Galpin, Joanne Meredith, Cathy Ure, Leslie Robinson

**Affiliations:** ^1^ Directorate of Psychology and Public Health School of Health Sciences University of Salford Salford United Kingdom; ^2^ Directorate of Radiography and Occupational Therapy School of Health Sciences University of Salford Salford United Kingdom

**Keywords:** decision making, eHealth, cancer screening, qualitative research, social media, mammography

## Abstract

**Background:**

The decision around whether to attend breast cancer screening can often involve making sense of confusing and contradictory information on its risks and benefits. The Word of Mouth Mammogram e-Network (WoMMeN) project was established to create a Web-based resource to support decision making regarding breast cancer screening. This paper presents data from our user-centered approach in engaging stakeholders (both health professionals and service users) in the design of this Web-based resource. Our novel approach involved creating a user design group within Facebook to allow them access to ongoing discussion between researchers, radiographers, and existing and potential service users.

**Objective:**

This study had two objectives. The first was to examine the utility of an online user design group for generating insight for the creation of Web-based health resources. We sought to explore the advantages and limitations of this approach. The second objective was to analyze what women want from a Web-based resource for breast cancer screening.

**Methods:**

We recruited a user design group on Facebook and conducted a survey within the group, asking questions about design considerations for a Web-based breast cancer screening hub. Although the membership of the Facebook group varied over time, there were 71 members in the Facebook group at the end point of analysis. We next conducted a framework analysis on 70 threads from Facebook and a thematic analysis on the 23 survey responses. We focused additionally on how the themes were discussed by the different stakeholders within the context of the design group.

**Results:**

Two major themes were found across both the Facebook discussion and the survey data: (1) the power of information and (2) the hub as a place for communication and support. Information was considered as empowering but also recognized as threatening. Communication and the sharing of experiences were deemed important, but there was also recognition of potential miscommunication within online discussion. Health professionals and service users expressed the same broad concerns, but there were subtle differences in their opinions. Importantly, the themes were triangulated between the Facebook discussions and the survey data, supporting the validity of an online user design group.

**Conclusions:**

Online user design groups afford a useful method for understanding stakeholder needs. In contrast to focus groups, they afford access to users from diverse geographical locations and traverse time constraints, allowing more follow-ups to responses. The use of Facebook provides a familiar and naturalistic setting for discussion. Although we acknowledge the limitations in the sample, this approach has allowed us to understand the views of stakeholders in the user-centered design of the WoMMeN hub for breast cancer screening.

## Introduction

### Background

Web-based tools provide significant opportunity to improve cancer-related health communication across the whole cancer spectrum, from prevention and screening to living with and beyond cancer [1]. Successful implementation requires an understanding of how the particular affordances of Web-based applications allow new opportunities for increasing health-related knowledge and decision making. It is also important to understand the particular informational needs and emotional experiences of the intended users. This paper presents the study conducted by the Word of Mouth Mammogram e-Network (WoMMeN) group to develop a Web-based resource to improve knowledge of and decision making in breast cancer screening. We focus in this paper on our analysis of an online *design group* who were brought together as a means of understanding the needs of our stakeholders.

In breast cancer screening, information on both benefits and risks needs to be balanced in order to help women make choices about whether to get a mammogram. This is a complex issue because the benefits are frequently disputed, and the risks, for example, treating a low-grade disease that was never going to develop into a cancer [2], can be devastating. These controversies are hotly debated in the medical field and the supporting evidence is contradictory. Understandably, women report being confused about whether to undergo screening for breast cancer [3] and uptake figures for breast cancer screening in the United Kingdom have steadily declined for 4 years up to 2015 [4]. Individuals using the Internet for electronic health (eHealth) must navigate a variety of information sources and weigh up the validity of the sources [5]. In the case of screening, they are required to apply this information to estimate the perceived risk, physical and emotional discomfort, inconvenience and usefulness of the screening, and the psychological and practical implications of detection [1].

Web-based tools offer the potential to facilitate decision making around screening by providing resources for communication, information, and shared experiences. In a related context, Skjøth et al [6] conducted qualitative research on the factors salient to care providers and pregnant women when considering screening for Down syndrome. Some of the women in the study reported a preference for resources on the Internet and advice from family and friends over the information booklets they received. They were keen to hear the experiences of pregnant women and placed importance on finding reliable information in a single location. These results highlight the desire to access both experiential information from women in a similar position (consistent with the rise in peer-to-peer health care) [7] and reliable information within a single resource. In this context, and other sensitive and complex health contexts such as breast cancer screening, it is important to understand how users access, consume, and respond to information before designing a Web-based resource. However, it is also necessary to seek the views of the health professionals who have a stake in ensuring that their service users are reliably informed.

The WoMMeN project was initiated through recognition of the potential for a digital resource to facilitate women in making informed decisions regarding breast cancer screening and to help them make sense of the potentially confusing data available. A project committee was established that included mammographers, psychologists, expert patients and service users, marketing and legal specialists, and a Web designer. This multidisciplinarity follows from recommendations for an integrated approach to eHealth tool development [8,9] and aligns our methods with the principles of user-centered design (UCD). The importance of UCD has been recognized in a number of approaches to eHealth decision aids that have sought to understand the needs of stakeholders and users through development [6,10]. The road map of the Center for eHealth Research and Disease Management (CeHRes) described by van Gemert-Pijnen et al [11] provides arguably the most comprehensive framework for applying UCD to eHealth product design. The CeHRes road map promotes (1) gaining an understanding of the lives of end users and other stakeholders (contextual inquiry), (2) seeking a deeper understanding of the values of key stakeholders (value specification), (3) involving users in the development of a product (design), (4) developing an operational plan for the implementation of the technology (operationalization), and (5) evaluating the product (summative evaluation).

The CeHRes framework is a useful lens through which to understand how we have involved stakeholders throughout the design of the WoMMeN hub (see Multimedia Appendix 1). We explored initial ideas through focus groups with potential service users (contextual inquiry) and from these emerged the idea that an online forum would meet women’s needs in seeking resources on mammography [12]. Potential features of the hub were ranked in importance in a modified card-sort by service users and practitioners. A beta version of the hub was developed and tested for usability issues with 6 service users (design), allowing tweaks before a wider launch. In addition, workshops have been run throughout the United Kingdom to address practitioners’ concerns with interacting online with clients [13] (operationalization).

### Objectives

The focus of this paper is on the novel approach we have taken to understand the key requirements, motivations, and anxieties of our stakeholders (the value specification phase in the CeHRes framework). In order to address difficulties in recruiting a face-to-face user design group from such a busy population, we decided to recruit a user design group through social media. This group was recruited in January 2015 and at the peak of the survey comprised 111 women (a roughly equal split of practitioners and nonhealth professionals). Members joined this closed Facebook group, which provided a naturalistic approach to understanding how women talked about breast cancer screening. The content from these conversations was analyzed to extract topics and values that underpinned how the hub was to be designed. To our knowledge, this is the first time that social media has been used in this way in the context of eHealth product design. Although we found this a supportive way of facilitating talk about breast cancer screening, we additionally wanted to supplement the approach by administering a more structured set of questions via an online survey posted to the Facebook group.

This paper therefore presents two complementary analyses that utilize both natural talk and survey data. The aim of this paper is to report the utility of our approach within a UCD context, so we present here a critical perspective of our data analysis using these methods.

## Methods

### Design

The wider project adopted a mixed-methods approach through the combination of qualitative data from the Facebook group and survey and quantitative data from the survey. The analysis presented in this paper is based on the qualitative thematic framework analysis that we conducted using data from both the Facebook group and survey.

### Participants

#### Facebook Group Participants

We took a pragmatic approach to recruitment. Each member of the research team, including practitioners, service users, and academics, used their own social media networks to advertise the project and recruit participants. To ensure we included the voice of a number of less well-represented groups, such as women with disabilities and women from black and ethnic minority groups, we also undertook more targeted recruitment via key informants from these groups who were known to us. However, we did not aim to stratify membership according to demographic information, and in this way, anyone was welcome to join. The only exclusions were men because of the potential that their inclusion may inhibit women in their discussions about breast health. There were 71 Facebook group members at the end point of the data sampling period.

#### Survey Participants

All Facebook group members were invited to take part in the survey. In total, 23 women participated; 12 were health professionals and 8 worked in breast cancer screening; 7 had received a cancer diagnosis and 6 had never had a mammogram.

### Survey Materials

Survey responses were collected using an 11-part anonymous survey distributed through Bristol Online Survey. The first nine sections concerned different aspects of the hub design: (1) topics of information, (2) organization of information, (3) search options, (4) communication options, (5) access to health professionals, (6) own posting preferences, (7) privacy and security, (8) regulation, and (9) additional features. Each main question was followed by a number of different options as to how a particular aspect might be designed, followed by free text boxes asking participants to “explain the decisions behind your ratings.” Question 10 was an additional free-text box asking whether there was anything else we had missed. Question 11 recorded professional background and mammography experience.

### Survey Procedure

An invitation to take part in the survey was posted on the Facebook group. An introductory screen informed participants of the purpose of the survey and assured them that any questions could be ignored. The survey took approximately 20 min to complete.

### Analysis

#### Facebook Data

All Facebook threads dated from February 2015 to July 2015 that related to breast cancer screening or the hub were extracted from the Facebook group. This amounted to 70 threads, with only those threads with more than 2 responses included in the analysis.

A total of 2 researchers independently analyzed the first 10 threads to identify topics of conversation. A consensus meeting was then held to construct the framework for analysis [14]. The framework comprised a number of themes identified during initial coding, and each theme was further divided according to group members’ background (service user, mammographer, WoMMeN research group member, and nonmammographer health practitioner). The remaining threads were then split between the researchers who each coded them based on the framework. Additional topic themes were noted, and a final consensus meeting was held to confirm theme saturation and to ensure new themes were embedded within the framework.

#### Survey Data

The qualitative data from the free-text survey responses was analyzed thematically according to the process described by Braun and Clarke [15].

## Results

The results of both the qualitative analysis from the Facebook group (denoted by thread number) and the analysis of qualitative answers to the survey (denoted by participant number) are presented here. In our analysis, we have also differentiated between health professionals (mammographers and health practitioners not working in breast cancer screening) and service users (nonhealth professionals who may or may not have had screening) to examine differences in stakeholder needs. From the data, two themes emerged: (1) the power of information and (2) the hub as a place for communication and support. In analyzing these themes, we hope to show the benefits of using an online user-design group.

### Theme 1: The Power of Information

In this theme, the importance of having balanced information on the hub was discussed. Women in both the survey and on Facebook suggested that it was important to have relevant, factual information that could embolden them to make clear and informed decisions.

Health professionals expressed the view that providing enough information is key to empowering service users to make decisions around breast cancer screening:

...knowledge Is power.

Facebook, thread 68, mammographer

I think it’s important to give women enough information about the screening process & examination so they are aware about what will happen when they attend.

Survey, p5, mammographer

The first quote comes from a mammographer on Facebook and was posted in response to a story about breast cancer death rates dropping in the United States. This initial message that “knowledge Is power” suggests that if women know that breast cancer screening may reduce rates of breast cancer death, they may be more likely to go for screening. In terms of designing the hub, then, having enough information about the right things is important; that is, not just the practical information but also information about why women should go for breast cancer screening. The second extract, also from a mammographer, further emphasizes that it is important for women to have enough information about the screening process. The fact that they suggest that women will need “enough” information about the process and examination implies that perhaps women do not always have this when they attend appointments.

However, we also found that some respondents highlighted that the information could potentially be misleading and threatening. One of the mammographers commented:

I think there is always scope to challenge/dispute/discuss what is reported in the media. Patients are so information hungry these days that we need to keep on top of what is being spread in the non-medical public domain to ensure its accuracy.

Facebook, thread 8, mammographer

This extract was posted on the Facebook group, and it orients to the fact that many people want a lot of information and will go to a variety of sites to gain this. She also notes, though, that there is a lot of inaccurate information in the public domain, particularly in the media, and staying aware of this information is important for practitioners.

However, health professionals do recognize that some women may prefer to avoid receiving too much information, as it can be overwhelming:

I appreciate many women are ostriches—that they would rather dig their head in the sand and not know until they have to.

Survey, p23, health practitioner

...some woman would be better off not knowing because once you know it’s there it will effect [sic] your quality of life for most woman and we are still not sure which is safe to leave and even then I’m not sure I would just leave it.

Facebook, thread 2, mammographer

The first example is a response to a question about what information women would like to see on the hub. The respondent suggests that some women would prefer not to have all the relevant information until they need to. The second example is slightly different, in that it comes from a Facebook discussion about women going for screening and finding precancerous cells, which might take many years to develop into cancer, if at all. Here the argument is made that for some women it would be better not to know about these precancerous cells.

Overall, the health professionals in our sample emphasized the importance of women receiving accurate information about breast cancer screening but also acknowledge that some women wish to limit the information they have access to. Health professionals plausibly have experience of, and a professional interest in, the ways in which women manage health information. Nevertheless, we found similar suggestions regarding information made by service users:

Knowledge of the whole process will help to allay fears.

Survey, p21, service user

This extract, from a woman with no experience of screening, supports the same view as the health professionals. She suggests that knowledge of the “whole process” is needed, which conceivably relates to the screening appointment, receiving results, what happens if you are recalled, and so on.

Service users also acknowledged the potential for information to be seen as a threat. The following extracts are both from women who had a cancer diagnosis:

Accuracy of mammograms—the statistics around breast cancer, risk factors, likelihood of its return, and the different types of breast cancer are mindblowing. In this sense I choose to limit how much information I seek out.

Survey, p16, service user

It would be better for them to be able to search for a particular area rather than having trawl through a lot of information and questions that may not be relevent [sic] to them at that time.

Survey, p21, service user

Here both the participants suggest that there is a huge amount of information available about breast cancer screening, and this can be overwhelming. Their cancer diagnoses may be relevant to this perspective as we would expect breast cancer screening information to have a particular emotional resonance. The service users’ extracts imply a desire for *control* over when and which information is accessed. This contrasts with the extract from the health professional suggesting women were “ostriches” who did not want to be exposed to some information.

Service users raised the issue of having access to patient stories, which was not prominent in the responses of health professionals:

Patient stories...positive and negative...are always powerful. When a professional wants to put info out there, personally think they should also be obliged to include case histories “for” and “against.”

Facebook, thread 8, service user

The poster argues that including patient stories on the hub can be helpful for users. This is, then, a different type of information, in that it is not merely information about the screening process or managing factual inaccuracies, but rather experiential information.

Analysis of the comments around information allowed some key principles to emerge to inform the design of the hub. Both the health professionals and service users recognized that although information is empowering, it can also be misleading or emotionally distressing. Health professionals more often emphasized the importance of factual materials, whereas service users called for experiential information. This highlights the need to provide a variety of sources for women on breast cancer screening, which is clearly indicated in this quote from a member of the research team:

This is why an on-line hub where women can have as little or as much as they want and in whatever format they want is better [then I would say that wouldn’t I!].

Facebook, thread 56, member of the research team

Our strategies for applying this evidence are described in the discussion.

### Theme 2: The Hub as a Place for Communication and Support

The second theme that emerged from the survey and Facebook data was that the hub should also be a place for communication between women on the issue of breast cancer screening and for women to be able to support one another. However, the type of communication emphasized differed between service users and health professionals. This is reflected in the first two extracts presented here, from a potential service user with no experience of breast cancer screening:

I think opportunities to communicate, share and support each other.

Survey, p4, potential service user

It’s invariably easier to deal with problems/concerns when you have someone/people in similar situations to turn and relate to.

Survey, p4, potential service user

This respondent suggests that an important part of the hub will be the chance for women who are invited to, and attend, breast cancer screening to support one another. A number of studies have noted the benefits of online forums in facilitating peer-to-peer support in symptomatic populations [16-18], and our results suggest this is also valued for asymptomatic populations making screening decisions.

Although lay people and service users were keen to emphasize support among peers, health professionals focused more on the potential for interaction between the screening population and practitioners:

I’m hoping that better quality information ad [sic] conversation going both ways from the women and the staff will help us all [...] we will at least be able to provide more support and information than we are able in the 6 short minutes available during the exam.

Facebook, thread 50, mammographer

I think it’s vital that health professionals be able to communicate with users in a variety of ways to suit their needs.

Survey, p3, mammographer

The first example suggests that practitioners often do not have enough time to speak in detail to women who go for screening. Therefore, having a Web-based resource could allow practitioners to achieve this without the time constraints of offline interactions. The second quote is from a mammographer in response to a question about how they would like to communicate with service users online. They are recognizing the need for a variety of routes for interaction, but it is not clear from the quote whether they are referring to the needs of the health professionals or the service users.

Despite both health professionals and service users being enthusiastic about the need to offer communication and support, there was also recognition of the potential pitfalls of doing this online and particularly in a text-only form of interaction:

I’d by concerned about inappropriate comments or misinterpreted dialogue.

Facebook, thread 6, service user

Virtual communication in an open community, existing without facial cues & intonation, will always present danger. It’s a bit like reading a novel, everyone’s experience is individual to how the reader interpreted the characters.

Survey, p3, mammographer

Discussions can get heated.

Survey, p11, mammographer

Participants noted a number of concerns about online communication, including inappropriate comments and the potential for arguments. Of particular concern was the lack of facial cues, which participants suggested might lead to misinterpretation of posts and, implicitly, to arguments. From the analysis, we noted that service users were interested in supporting each other, whereas health professionals were interested in supporting service users. Therefore, the hub should provide a way for both service users and health professionals to communicate with each other.

## Discussion

### Principal Findings

In this research, we sought to use a novel method to inform the design of a Web-based resource to aid decision making around breast cancer screening. We drew on the CeHRes framework [11] to inform our methodology, and we have reported here how we addressed the *value specification* of stakeholders through a social media–based user design group. This approach allowed us to involve users in the design of a resource for breast cancer screening through analyzing the comments within a Facebook group, in addition to survey responses.

Our findings showed that women want both information and support around decision making in breast cancer screening. Health professionals and service users showed the same broad concerns overall. However, there were subtle differences in the way these were expressed, revealing potentially different needs in a Web-based resource. This is highlighted, for instance, through the emphasis on the health professionals’ concern over accurate information provision and the service users’ focus on experiential information and control over information consumption. Therefore, the design of the hub was influenced by these different needs. As both service users and health professionals valued access to accurate information, all information posted on the site is curated for accuracy by experienced mammographers. We suggest that any health resource seeking buy-in from health professionals should acknowledge their stake in managing the misleading information that may exist in the public domain. Our findings that both health professionals and service users recognized the need for choices around what information is accessed led us to incorporate different types of information on the hub in distinct areas. For example, the breast cancer screening process was mentioned by both groups of stakeholders, and therefore, we have included a distinct area within the hub describing the mammogram. We have also included tabs for general information, frequently asked questions, and a research area for women who want to access original papers. Service users valued experiential information, and this is supported on the hub through a blog and forum so women can access and share a range of experiences. The blog and forum shared a dual purpose. They allowed women to interact regarding their experiences, which the service users in particular suggested as important. The forum also allowed practitioners to engage in discussion and provide information or to point women to sources of accurate information. Women’s concerns about the potential issues regarding discussions becoming heated are managed through the forum being moderated by members of the WoMMeN research team. There is also a *pinned* ethical statement at the entry point to the forum, which reminds posters of their ethical obligation to respect other people’s views and sensitivities.

One benefit of having a user group that includes people who are *naïve* participants is that they may think about aspects of an online group, such as peer-to-peer support, which health professionals and researchers might conceivably consider a lower priority relative to factual information. However, the downside of having users with no experience of, in this case, breast cancer screening is that perhaps they will not understand precisely what issues may arise from that process and so their responses may not come from experience. Our approach is evidence that stakeholders with different levels of domain expertise can be engaged in online dialogue together to produce useful insights into their particular needs.

One of the strengths of our approach was how the Facebook group data and the directed survey questions compensated for the limitations of each method. The survey allowed for direct questions to be asked of the group, but surveys are also “inherently limited by the questions they ask” [19]. By also using the Facebook group, it meant we were not constrained to just ask direct questions, but we could also draw upon naturally emerging topics of conversation. There are a number of benefits to using more naturalistic data in these contexts; they allow for novel questions and issues that are of interest to the participants to be raised, and they reduce the role of the researcher in the interaction [20]. The Facebook group also meant that the members of the user design group were not constrained by time and space, and so they could engage in the group at a time of their choosing and in the comfort of their own home [21]. It also meant that we could take a more longitudinal approach when consulting with our participants about design choices. The Facebook group, however, was not anonymous. Members of the research team posted in that group, and their presence could potentially have limited discussions. Therefore, the survey allowed us to create an anonymous space for respondents to indicate what they wanted in the hub.

A second benefit of the online design group was the ability to triangulate our findings from both types of data [22]. The naturally occurring discussion in the Facebook group often supported the comments that were made when asked directly through the survey. For instance, women in the survey expressed their apprehension about misinterpreted dialogue, and this was also raised naturalistically in the Facebook group where the issues of the lack of interpretation and facial cues were discussed. This suggests it was a *natural* concern of participants rather than just one that participants raised when questioned. It is noteworthy that such comments came from the Facebook group where individuals were interacting with relatively little heated debate, although there were, of course, disagreements. In fact, we saw participants providing each other with support when group members went for mammograms (eg, “thanks for letting us all share your mammogram experience virtually”). In general, the lack of prominent differences between the two datasets was an interesting feature of our findings.

### Limitations

One important consideration is the characteristics of participants in a social media–based design group. Individuals who sign up to research are often highly motivated and knowledgeable [23,24] and as such there may be self-selection bias. The women in the Facebook group had often had experience of breast cancer or were health professionals and were particularly health literate. That they were therefore motivated by the topic and generally positive about the importance of attending breast cancer screening may mean that they were not typical of women who are invited for breast cancer screening. Therefore, when using these methods of user design, it is important to take account of the fact that many of the people involved in a user design group may actually be very motivated and knowledgeable. An implication is that they may sometimes make decisions about what they think should be on the hub based on what *women in general* wanted rather than what they, as motivated, knowledgeable women wanted. However, this issue is not exclusive to online research and also affects offline patient and public involvement groups [25]. What might be problematic for our particular approach is whether the level of Internet literacy of our group is reflective of everyone invited for breast cancer screening and, in particular, the women over 50 who may not use Facebook or other social media [26].

A further limitation involves the presence of the research team within the Facebook design group. It could be argued that this potentially impacted on how free the women in the group felt to be able to voice negative opinions about the hub. However, each individual, including those from the research team, has multiple identities in relation to the topic. For instance, a member of the research team could also have experience of being screened, of having cancer, and of being a health professional. Therefore, within the group, they were not always acting as a member of the research team but brought their own experiences to the discussions. This, along with off-topic posts (eg, sharing recipes or cultural topics), potentially reduced the salience of the group as a research context and the research team as researchers.

### Conclusions

The data have enabled us to create the WoMMeN hub with features women told us they (and other women) wanted. Our analysis allowed us to see that information and support are valued within the context of breast cancer screening by both health professionals and service users. However, by also acknowledging the orientation of the respondents, differences in the way they prioritized these emerged. Web-based decision aids provide valuable opportunities to empower service users. However, to facilitate their success, our data suggest they should embed opportunities for experts to dispel misleading information while allowing service users to exchange experiences. Therefore, our work has shown that it is essential to understand that *one size does not fit all* and designers need to be aware of the requirements of specific stakeholders through a user-centered, participatory approach. The methods we have reported here may help in this regard by providing a convenient and accessible online environment in which insight can be gained from natural dialogue and validated by direct questioning.

## Appendix

Multimedia Appendix 1 A PDF document containing screenshots of the Wommen Hub.

## Abbreviations

CeHRes: Center for eHealth Research and Disease Management
eHealth: electronic health
UCD: user-centered design
