# Supplementary material for: “Thanks for Letting Us All Share Your Mammogram Experience Virtually”: Developing a Web-Based Hub for Breast Cancer Screening
Source: JMIR Cancer. 2017 Oct 27;3(2):e17. doi: 10.2196/cancer.8150 (PMC5681724; doi:10.2196/cancer.8150)
Supplement: Multimedia Appendix 1 [file cancer_v3i2e17_app1.pdf]

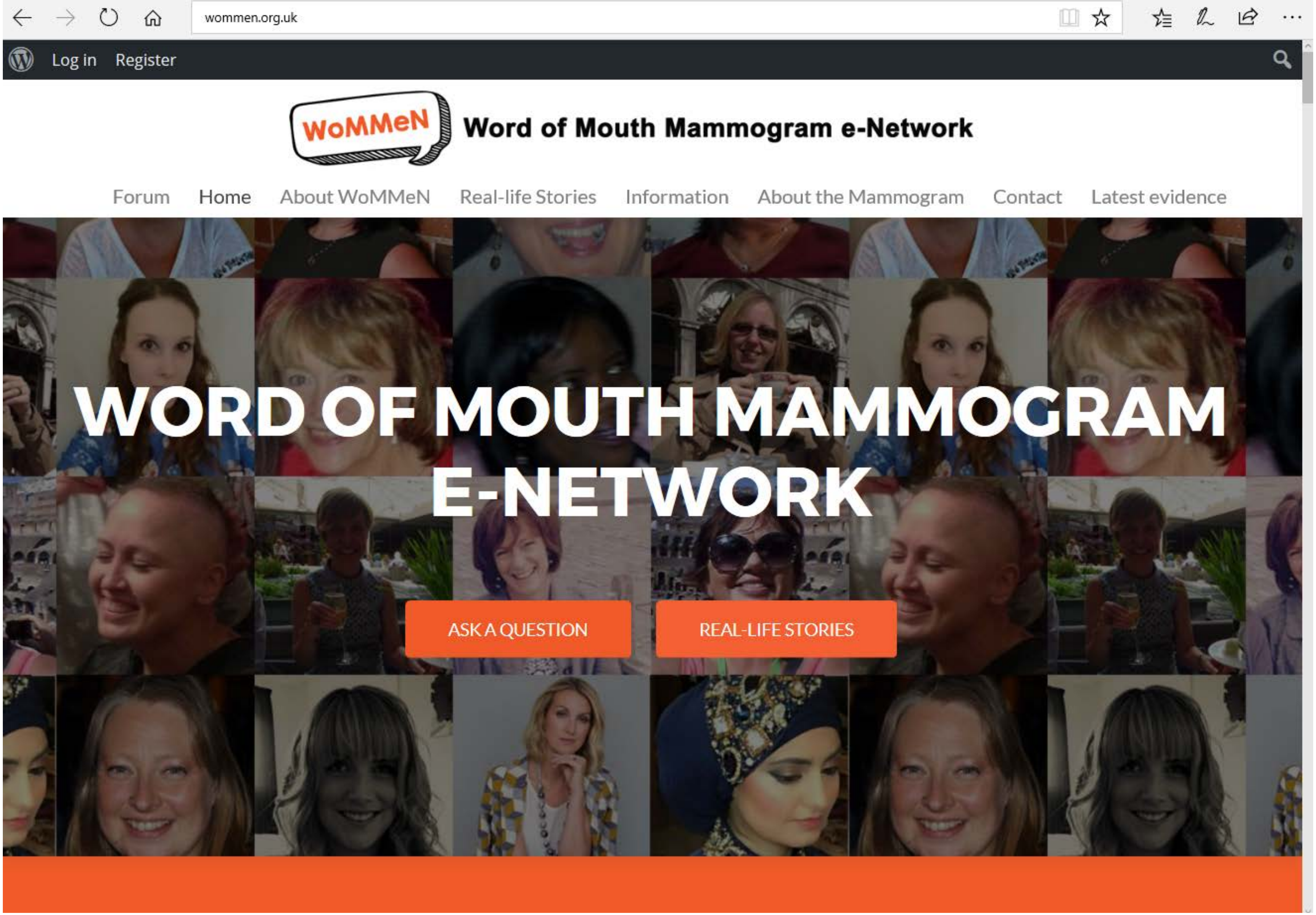
wommen.org.uk
Log in
Register
WoMMeN
Word of Mouth Mammogram e-Network
Forum
Home
About WoMMeN
Real-life Stories
Information
About the Mammogram
Contact
Latest evidence
WORD OF MOUTH MAMMOGRAM E-NETWORK
ASK A QUESTION
REAL-LIFE STORIES

About the Mammogram

Here are some frequently asked questions about the mammogram. There's more detailed information elsewhere on the site – check out recent posts on our blog here or ask a question on our forum here

The Procedure

Who performs the mammogram?
A female health practitioner who has specialised in breast imaging. They work alone or sometimes in a pair. All practitioners have qualifications to practice mammography.

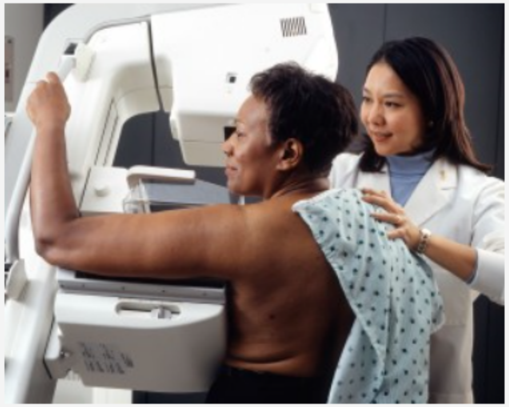

Will the staff always be female?
In the UK National Breast Screening Service the imaging staff who will perform your mammogram are all female.

Can I be screened if I have breast implants?
Yes you can, please let the screening unit know in advance as extra time is allocated for your appointment. The mammograms will only show the breast tissue surrounding the implant.

LOGIN

Username

Password

☐ Remember Me

LOG IN Register

TAG CLOUD

Asian assessment

wommen.org.uk/research-2

Log in Register

WoMMeN Word of Mouth Mammogram e-Network

Forum | Home | About WoMMeN | Real-life Stories | Information | About the Mammogram | Contact | Latest evidence

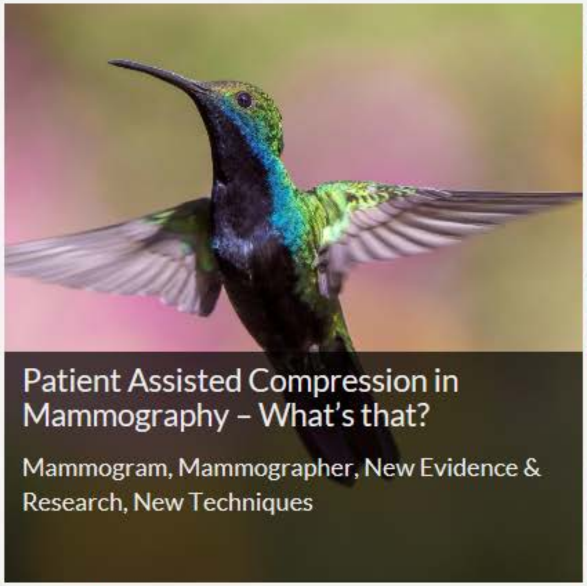

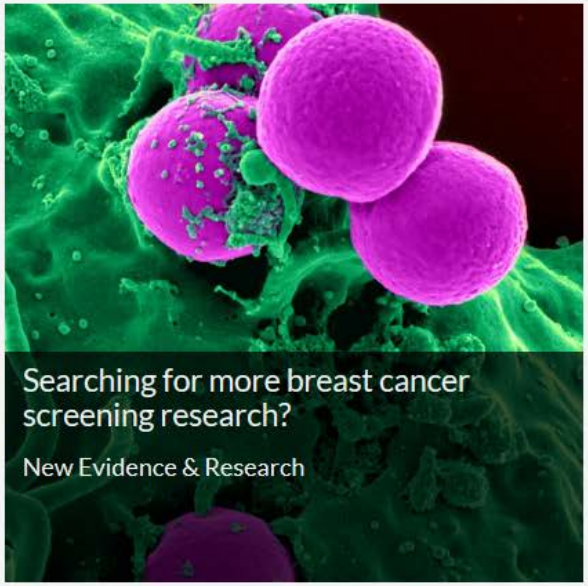

England

NHS Breast Screening Programme

TAG CLOUD

Asian assessment BME Breast cancer breast cancer awarness breast culture Breast density breast examination breast health breast implants breasts breast screening breast screening

"The Mammographer was brilliant"

Posted on *August 21, 2017* by *Leslie*

It's great to get patient stories and here's one to encourage those of you who are anxious about going for your breast screen. Thanks to Jean Phillips for sharing this with us and telling us how kind the mammographer was.

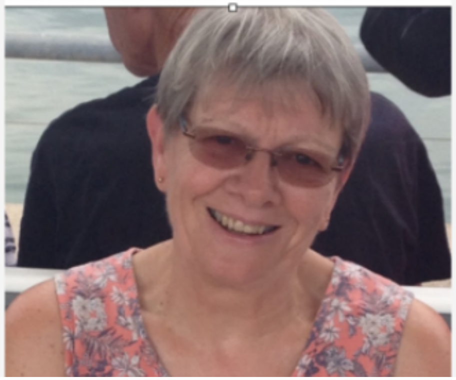

I've recently been for a Mammogram at Nye Bevan House, Rochdale. It was a routine check, the kind that I've been having every 3 years since I was 50 (I'm now 65). I set off from home feeling really brave but after arriving and sitting in the waiting room for a while I could feel my anxiety rising. I got into a conversation with a couple of ladies and we were all of the same opinion. It's horrible, it's embarrassing, it's unpleasant, it hurts, it's uncomfortable, it's scary etc etc. My name was called along with another lady and we moved further up the corridor. I went in first and half jokingly

LOGIN

Username

Password

☐ Remember Me

LOG IN Register

TAG CLOUD

Asian assessment

wommen.org.uk/forums/topic/consideration-in-communication

Log in Register

WoMMeN **Word of Mouth Mammogram e-Network**

Viewing 1 post (of 1 total)

Author	Posts

December 15, 2015 at 9:19 pm #465

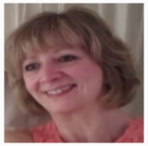

Leslie

Keymaster

Like 4

A quick reminder about some issues related to sharing information and opinions in an on-line social space. This is an open forum. That means that anyone can read the posts. You do have to register to add posts or join the conversation however. On joining you are asked to agree to a set of principles before you post. Although we welcome open discussion and encourage the sharing of different opinions, you should never post anything that aims to deliberately upset other people. The administrators will remove posts that are likely to cause offense or harm.

The practitioners present on this forum share their knowledge and expertise freely because of their passion to help women understand more about breast screening. In doing so they are not acting as employees but as autonomous practitioners with a wide range of experience which they are keen to share with you. For this reason they can not, nor should/will they, offer advice about individual cases. If you are concerned at all about your own health or that of a friend or relative you should seek advice from your GP.

This topic was modified 1 year, 10 months ago by Leslie.

Password

Remember Me

LOG IN Register

TAG CLOUD

Asian assessment BME Breast cancer breast cancer awarness breast culture Breast density breast examination
